# Impact of Personality Features and Interpersonal Problems on Anxiety Among Older Adults

**DOI:** 10.1093/geroni/igab046.3238

**Published:** 2021-12-17

**Authors:** Olivia Noel, Daniel Segal, Katie Granier, Marissa Pifer, Lisa Stone

**Affiliations:** 1 Peak View Behavioral Health, Colorado Springs, Colorado, United States; 2 University of Colorado at Colorado Springs, University of Colorado at Colorado Springs, Colorado, United States; 3 University of Colorado Colorado Springs, Colorado Springs, Colorado, United States

## Abstract

Introduction: Anxiety is a significant mental health problem among older adults and is associated with multiple other mental disorders, poor psychosocial functioning, and reduced quality of life. Personality traits and disorders, along with interpersonal problems, may play a significant role in anxiety, but these relationships are not well understood among older adults. This study examined relationships between anxiety with normative personality traits, personality disorder (PD) features, and interpersonal problems. Method: Community-dwelling older adults (N = 130) completed the Geriatric Anxiety Scale (GAS), Coolidge Axis Two Inventory (CATI), Big Five Inventory-2 (BFI-2), and Circumplex Scales of Interpersonal Problems (CSIP). Results: Anxiety was positively correlated with 13 of 14 CATI PD scales, ranging from .23 (Narcissistic) to .61 (Depressive). Regarding normative personality, anxiety was associated with Agreeableness (-.23), Conscientiousness (-.30), Extraversion (-.31), and Negative Emotionality (.56). Regarding interpersonal problems, anxiety was positively related to all eight CSIP scales: Self-Sacrificing (.30), Domineering (.31), Exploitable (.40), Intrusive (.41), Self-centered (.47), Nonassertive (.50), Socially Inhibited (.60), and Distant/Cold (.62). Regression analyses indicated that PD features accounted for the most variance in anxiety (53%), followed by interpersonal problems, (46%) and normative personality traits (33%). Discussion: Anxiety appears to be meaningfully associated with PD features, several aspects of normative personality, and interpersonal problems, suggesting that these variables may play a role in the development of anxiety, or vice versa. Our findings especially speak to the growing awareness of the deleterious impact of PD features on clinical syndromes in later life, as evidenced by strong comorbidities with anxiety.

